# Elevated CO_2_, warming and drought differentially impact reproductive and vegetative economic traits in two grassland species

**DOI:** 10.1093/aob/mcaf214

**Published:** 2025-09-10

**Authors:** Murugash Manavalan, Dinesh Thakur, Andreas Schaumberger, Michael Bahn, Zuzana Münzbergová

**Affiliations:** Department of Botany, Faculty of Science, Charles University, Prague 128 01, Czech Republic; Institute of Botany, Academy of Sciences of the Czech Republic, Průhonice 252 43, Czech Republic; Institute of Botany, Academy of Sciences of the Czech Republic, Průhonice 252 43, Czech Republic; Institute of Plant Production and Cultural Landscape, Agricultural Research and Education Centre Raumberg-Gumpenstein, Irdning-Donnersbachtal 8952, Austria; Department of Ecology, Universität Innsbruck, Innsbruck A-6020, Austria; Department of Botany, Faculty of Science, Charles University, Prague 128 01, Czech Republic; Institute of Botany, Academy of Sciences of the Czech Republic, Průhonice 252 43, Czech Republic

**Keywords:** Floral economic spectrum, plant economic spectrum, functional ecology, ecological strategies, reproductive success, trade-off, *Lotus corniculatus*, *Crepis capillaris*

## Abstract

**Background and Aims:**

Since the Industrial Revolution, rising atmospheric CO_2_, warming and more frequent droughts have significantly impacted ecosystems. While the responses of leaf functional traits to these climate change factors have been widely studied, reproductive traits remain relatively understudied, despite their key role in the diversification and distribution of flowering plants. Here we investigated how elevated CO_2_, warming, drought and their interactions affect floral, leaf and seed traits in two model grassland species. We also examined how these factors influence trait coordination.

**Methods:**

Two common grassland species, *Lotus corniculatus* and *Crepis capillaris*, were sampled from a 10-year climate manipulation experiment. We measured resource economic traits related to organ size, construction cost and dry matter content in both leaves and flowers, along with seed size and number. Univariate and multivariate analyses were used to assess trait responses, and rank–abundance curves were employed to visualize changes in trait coordination across treatments.

**Key Results:**

Trait responses to climate change factors varied between species. Drought emerged as the most influential factor, affecting only leaf traits in *L. corniculatus*, but impacting leaf, floral and seed traits in *C. capillaris*. Across both species, climate change conditions increased leaf construction costs and reduced flower size. In addition, it led to larger leaves in *L. corniculatus* and fewer seeds in *C. capillaris*. Under extreme climate change conditions, trait coordination became stronger in both species, although *C. capillaris* showed no coordination response specifically to drought.

**Conclusions:**

Our results show that floral economic traits, like leaf traits, are responsive to individual and combined effects of climate change factors. This highlights their importance in shaping plant strategies under environmental stress and emphasizes the need to better integrate floral traits into the whole-plant economic framework.

## INTRODUCTION

Human activities since the Industrial Revolution have significantly increased greenhouse gas emissions, leading to today’s high levels of atmospheric CO_2_, which is the main driver of the ongoing climate change (Borghi *et al.*, 2019; IPCC, 2022). The direct and indirect effects of this change have resulted in rapid alterations of functioning of plants and entire natural ecosystems (Feehan *et al.*, 2009). Plants form the foundation of natural ecosystems, serving as primary producers that sustain all other life forms by driving energy flow and nutrient cycling. Due to the significant impact of climate change on ecosystems, it becomes crucial to understand the effects of different climate change factors such as CO_2_, warming and drought on plants (Descamps *et al.*, 2021*b*). Such knowledge can help to predict and mitigate the adverse effects of climate change on biodiversity and ecosystem services, ensuring the sustainability and resilience of natural ecosystems.

Plant functional traits have long been used by ecologists as useful proxies of plant ecological strategies (Wright *et al.*, 2004; Reich, 2014) to gain insights into species response to changing environmental conditions (Liu *et al.*, 2024; Tyagi and Kumar, 2024). Recent major climate change factors, particularly elevated CO_2_, temperature and drought, significantly impact plant traits and physiology across multiple scales, although to different extents (Lukac *et al.*, 2010; Albert *et al.*, 2011; Bjorkman *et al.*, 2018; Abdelhakim *et al.*, 2022). Drought, due to its ease of implementation in experimental studies, seems to be the most extensively studied of all the climate change factors. Among the climatic factors, it produces the most uniform and predictable negative response in a range of plant functional traits, including vegetative traits related to competitive ability (plant height and leaf area) (Lidon, 2012; Burkle and Runyon, 2016; Zhou *et al.*, 2018; Rodríguez-Alarcón *et al.*, 2022), resource use strategy traits such as specific leaf area and specific root length (Jung *et al.*, 2014; Wellstein *et al.*, 2017; Rodríguez-Alarcón *et al.*, 2022), and reproductive traits such as flower size and number, floral rewards and seed set (Kuppler *et al.*, 2021; Akter and Klečka, 2022; Höfer *et al.*, 2023).

In contrast, the response of plant traits to factors such as temperature and CO_2_ are more inconsistent and context-dependent, varying by species, traits and environmental conditions. While warming has been shown to have significant negative effects on both vegetative and reproductive traits (Niu *et al.*, 2000; Liang, 2016; Descamps *et al.*, 2021*b*), studies have revealed that traits such as leaf area, stem length, stem diameter, fine-root biomass and pollen germination are optimized at a certain temperature (Nicotra *et al.*, 2008; Dostálek *et al.*, 2020; Wang *et al.*, 2021; Shin *et al.*, 2023; Kumarathunge *et al.*, 2024) and decline with both temperature increase and decrease. Due to the variability of temperature optima across species, the effects of temperature on plants are much more species-specific compared with the more consistent effects of drought.

Elevated CO_2_ generally has a positive effect on plants, enhancing photosynthesis, leaf area and carbohydrate concentration and boosting plant biomass when water is sufficient (Duan *et al.*, 2018; Temme *et al.*, 2019). Long-term free-air CO_2_ enrichment (FACE) experiments have further validated these findings, showing sustained increases in photosynthetic rates and biomass accumulation over several growing seasons (Ainsworth and Long, 2005; Leakey *et al.*, 2009). However, the effects of elevated CO_2_ on plant reproductive traits are far less studied compared with its effects on vegetative traits (but see Wang *et al.*, 2015; Glenny *et al.*, 2018).

While numerous studies have examined the individual effects of temperature, moisture and elevated CO_2_ on plant traits, these environmental factors often interact in complex and non-additive ways. This has led to a growing emphasis on investigating their combined effects, particularly on leaf morphology (e.g. Qaderi *et al.*, 2006; Albert *et al.*, 2011; Mueller *et al.*, 2022). However, the interactive impacts of these climate change drivers on reproductive traits, especially in relation to resource allocation strategies, remain largely understudied.

Understanding how plants allocate resources under stress is central to predicting their survival and reproductive success in changing environments. The concept of plant economic spectra provides a valuable framework for this, linking traits such as leaf thickness, tissue density and dry matter content to construction costs and resource-use strategies (Wright *et al.*, 2004; Freschet *et al.*, 2010; Díaz *et al.*, 2016). While the leaf economic spectrum has been widely studied (Wright *et al.*, 2004; Reich, 2014), recent work has extended this framework to reproductive structures through the emerging concept of the floral economic spectrum (Roddy *et al.*, 2021; E-Vojtkó *et al.*, 2022). This approach identifies key trade-offs in floral trait construction and function, offering insights into how plants balance investment in reproduction with environmental constraints.

Studying floral economic traits under climate change conditions is particularly important, as flowers are critical for reproductive success and pollinator interactions (Ceulemans *et al.*, 2017). Environmental stresses such as drought and warming have been shown to affect flower size as well as nectar production and composition, which in turn influence pollinator visitation patterns (Descamps *et al.*, 2021*b*; Martén-Rodríguez *et al*., 2025). These stresses may also affect the energy and carbon resources that plants allocate in building and maintaining floral tissues, often reflected in shifts related to construction cost-related traits. By examining how floral economic traits respond to environmental stressors, we can better understand the adaptive strategies plants use to optimize reproductive investment and anticipate how plant–pollinator dynamics and ecosystem functions may shift under future climate scenarios.

Most studies examining the response of plant traits to environmental factors focus on the variation of traits in individual plant organs (e.g. Nie *et al.*, 2013; Jung *et al.*, 2014; Bongers *et al.*, 2017; Hai *et al.*, 2023; Fenollosa *et al.*, 2024). However, different organs respond to environmental factors in distinct ways, and their responses may also covary. For example, an analysis of leaf and root traits across 24 species found that leaf traits exhibited stronger and more homogeneous responses to drought, while root trait responses were highly variable and species-specific (Lozano *et al.*, 2020). This highlights the need to investigate how traits across different organs coordinate under stress. While floral traits are often considered decoupled from leaf traits due to differing selective pressures (Salguero-Gómez *et al.*, 2016; E-Vojtkó *et al.*, 2022), emerging evidence suggests that coordination does occur. For instance, recent findings show correlations between floral traits such as pollen grain number and nectar volume with leaf traits such as leaf area and leaf dry matter content, indicating that trait integration across organs may be more common than previously assumed and warrants further investigation (Fantinato *et al.*, 2025).

The present study seeks to fill the above-identified gaps in our knowledge by investigating how climate change factors, namely CO_2_, temperature and drought, affect floral, leaf and seed traits and the covariation between them in two model grassland species. Specifically, this study addresses the following questions and hypotheses.

Question 1. How do floral, leaf and seed traits vary in response to elevated CO_2_, warming and drought?

Hypothesis 1. Drought will induce the most consistent and negative changes across plant traits, whereas elevated CO₂ is expected to have more positive effects due to enhanced photosynthetic activity.

Question 2. How do these climate change factors interact to affect floral, leaf and seed traits?

Hypothesis 2. In the interaction between temperature and CO_2_, CO_2_ would negate the negative effects of warming. Increasing temperature would exacerbate the negative effects of drought, which would be partially mitigated by elevated CO_2_.

Question 3. How do climate change factors influence the covariation of flower, leaf and seed traits?

Hypothesis 3. Elevated climate change factors exert a strong filtering action on the covariation of plant traits across different organs, leading to coordinated trait adjustments to adapt to stressful conditions. Similar to leaf traits, floral traits would exhibit adaptive changes in response to individual and combined climate change factors, and these changes would be strongly coordinated with leaf and seed trait responses.

## MATERIALS AND METHODS

### Study site and experimental setup

This study was conducted within the framework of a long-term multifactorial climate change experiment (named ClimGrass) at the Agricultural Research and Education Centre (AREC) in Raumberg-Gumpenstein (Meeran *et al.*, 2021; Reinthaler *et al.*, 2021; Radolinski *et al.*, 2025). This study site is located in Styria, Austria (47°29′37″N, 14°06′0″E), 710 m above sea level, and was established in 2012 in a managed submontane C3 grassland. The soil is classified as Cambisol with loamy texture. Above-ground biomass is mown and removed three times a year and plots are regularly bolstered with mineral fertilizer with a total load of 90 kg N ha^−1^ year^−1^, 65 kg P ha^−1^ year^−1^ and 170 kg K ha^−1^ year^−1^. This is done to compensate for the macronutrients lost due to harvests (Pötsch *et al.*, 2020). The experimental design comprises a total of 54 plots (4 × 4 m each) representing individual and combined effects of three levels of temperature (ambient, +1.5 °C, +3 °C), atmospheric CO_2_ concentration (ambient, +150 ppm, +300 ppm) and drought (yes/no) (Piepho *et al.*, 2017; Pötsch *et al.*, 2020). The CO_2_ and temperature treatments started in May 2014 and the drought treatments started in May 2017. The different temperature and CO_2_ levels were established based on potential future climate scenarios and were simulated through a combination of infrared heating systems to increase air temperature and a mini-FACE approach for fumigation with CO_2_ (Piepho *et al.*, 2017; Pötsch *et al.*, 2020). Dynamic rainout shelters controlled by rain sensors were deployed on 12 plots to generate water stress in combination with ambient and future climate change. These shelters were only used to restrict rain and induce drought during the summer seasons (exact dates are provided in Supplementary Data Table S8). Half of the drought plots were maintained at ambient conditions with the remaining half being exposed to the extremes of CO_2_ (+300 ppm) and temperature(+3 °C) (Radolinski *et al.*, 2025; Tissink *et al.*, 2025). The plots that acted as control were not heated or fumigated and were equipped with non-functional heaters and/or mini-FACE rings of the same shape and size which blew ambient air to account for potential disturbances (Piepho *et al.*, 2017; Pötsch *et al.*, 2020)

### Plant sampling and trait measurements

For this study, we selected a set of plots representing six treatments: (1) control (C0T0D0); (2) elevated CO_2_ concentration (+300 ppm) (C1T0D0); (3) increased temperature/warming (+3 °C) (C0T1D0); (4) drought (C0T0D1); (5) combination of elevated CO_2_ and warming (C1T1D0); and (6) combination of elevated CO_2_, warming and drought (C1T1D1). Each of these treatments was represented by at least four plots, providing consistent replication across the manipulated climate conditions. The plots were additionally narrowed down based on the presence of either of two model grassland species, *Lotus corniculatus* and *Crepis capillaris*. These two species are the two most common insect-pollinated species occurring in the experimental plots and have the same flowering period.

*Lotus corniculatus* (Fabaceae) is a polycarpic perennial non-clonal herb widely distributed across Europe (Chytrý *et al.*, 2021). It flowers in an umbel inflorescence type (Chytrý *et al.*, 2021). The corolla is zygomorphic, yellow, and consists of five petals: the upstanding ‘standard’, the lateral two ‘wings’ and the final two lower petals uniting to form the ‘keel’, the overall shape of the corolla being butterfly-like (Nature Gate, 2011). The plant is primarily insect-pollinated.

*Crepis capillaris* (Asteraceae) is an annual herb and is native in Europe, being widespread as far north as Denmark and southern Sweden (Stace, 2010; Govaerts, 2024). It flowers in a synflorescence corymbiform inflorescence type. Each flower head contains 50–70 flowers. The corolla is ligulate, 6.0–15.0 mm long, and yellow with a ligule tinged red (Kilian, 2024). It is pollinated by insects but is also capable of selfing (Chytrý *et al.*, 2021).

The sampling was conducted on 22 and 23 July 2023. For *L. corniculatus*, a total of 39 individuals were sampled from 28 plots across the six treatments, with 5–11 individuals sampled per treatment. From each individual, one to four flowers and one or two leaves were collected. For *C. capillaris*, 45 individuals were sampled from a total of 21 plots with six to ten individuals per treatment. From each *C. capillaris* individual, one or two flowers and leaves were sampled (exact numbers of individuals and plots sampled for each species and treatment are detailed in Supplementary Data Table S1). The variation in the number of individuals, leaves and flowers depended on their availability in the different treatments (selecting only fully developed but not wilted and undamaged leaves and flowers/inflorescences). Within each plot, samples were collected at least 10 cm apart with a maximum of two samples per plot, wrapped in water-saturated paper towels and stored in the refrigerator until further processing (∼48 h). Alongside flowers and leaves of both species, seeds of *C. capillaris* were also sampled, dried in paper bags and stored. Developed seeds were not available in *L. corniculatus* and thus could not be sampled.

To estimate leaf area (LA), specific leaf area (SLA) and leaf dry matter content (LDMC), each leaf, which had previously been wrapped in a wet paper towel for at least 48 h (during sampling), was weighed to determine its saturated mass and then subsequently scanned in a flatbed scanner to determine the LA. The leaves were dried at 70 °C for 72 h, after which their dry mass was also taken. Leaf area was estimated using ImageJ software. Specific leaf area was calculated as LA divided by leaf dry mass and LDMC was estimated as the ratio of leaf dry mass to saturated mass (Pérez-Harguindeguy *et al.*, 2013).

The flowers of the two species were processed differently due to variation in their morphology. The petals of *L. corniculatus* were dissected out from the flowers and weighed to obtain their saturated mass. Afterwards, they were separated out into their individual petal types: one standard petal, two wings and one keel. The separated petals were then flattened and scanned, dried similarly to leaves and then weighed again using a Mettler Toledo Microbalance (*d* = 0.5 μg) to get their dry mass. The scanned petals were processed through ImageJ to obtain their individual petal area. As the keel is composed of two fused petals, its area was multiplied by 2. The total petal area of all the petals was taken as the display area (DA). The total specific petal area (SPA) of the whole corolla of each flower was calculated (DA/dry mass). Petal dry matter content (PDMC) was calculated by dividing the total dry mass of the petals by their saturated mass (similarly to leaves, the petals had been wrapped in wet tissue for at least 48 h prior estimating saturated mass).

For *C. capillaris*, five individual ray florets were dissected out and then weighed, flattened and scanned to get their fresh mass and petal area. They were then dried and weighed to get their dry mass. Due to the homogeneity of ray florets, the florets were processed together, and their SPA was calculated as the total area of five ray florets divided by their total dry mass. Here, DA was calculated as an average of the five individual ray florets. Petal dry matter content was also obtained. The number of seeds in each flower head was counted. Seed number per flower head (SN) was calculated by dividing the total number of seeds taken from a plant by the number of flower heads. The total weight of the seeds in a flower head was determined using the microbalance mentioned above. Seed mass (SM) was then calculated by dividing the weight of all the seeds in a flower head by the number of seeds.

### Statistical analyses

All statistical analyses were carried out in R software version 4.3.1. AI assistance was used to generate initial R code for certain analytical approaches. Two separate datasets were prepared for the univariate and multivariate analyses. For the univariate analysis, trait values from leaves or flowers of the same individual were retained as separate data points. In contrast, for the multivariate analysis trait values were averaged per individual prior to analysis. Prior to multivariate analyses, all traits were standardized to a mean of 0 and a standard deviation of 1 to account for differences in measurement scales across traits.

To assess the broad patterns of response of different functional traits to climate change factors, a redundancy analysis (RDA) was first performed using the vegan package in R (version 2.6.10; Oksanen *et al.*, 2025) followed by a permutational analysis of variance (PERMANOVA) using 9999 permutations to test the significance of the climate change factors in the RDA.

To assess the impact of the climate change factors on individual plant traits, we conducted a set of univariate analyses where first we performed a Shapiro–Wilk test to evaluate the normality of the trait values. Based on the *P*-values obtained, the normality of the original trait value was compared with its logarithmic and square-root transformations. The form of the trait value with the highest *P*-value was selected for inclusion in the linear models, as it indicated the closest approximation to a normal distribution. In *L. corniculatus*, SPA and LDMC were square-root- and log-transformed respectively while all other variables were used in their original form. In *C. capillaris*, only DA was kept in its original form, with SPA, PDMC, LDMC and SN being square-root-transformed, and LA, SLA and SM being log-transformed. Linear models were used to analyse the effects of CO_2_ (C), temperature (T), drought (D) and their interactions (CO_2_ × temperature (C × T) and (CO_2_ + temperature) × drought (CT × D)) on leaf and reproductive traits of both species separately. All environmental variables and their interactions were included simultaneously as fixed predictors within a single model, without applying variable selection procedures such as stepwise or backward elimination. The ggplot2 package (version 3.5.1) (Wickham, 2016) was used for creating the plots.

To understand general patterns of covariation across all plant traits, principal component analysis (PCA) was conducted and visualized using the factoextra package (version 1.0.7) (Kassambara and Mundt, 2020) in R.

Pairwise correlations between individual plant traits were analysed by means of a trait correlation network constructed using significant Pearson correlations. A threshold of *r* > 0.2 marked pairwise correlations that were significant at *P* < 0.05. All correlations below this threshold were set to 0, yielding the adjacency matrix **A** = [*a_i_*_,*j*_] with *a_i_*_,*j*_ ɛ [0,1]. Additionally, network connections between any pair of traits are weighted by the absolute correlation strength, |*r_ij_*| (Langfelder and Horvath, 2008; Kleyer *et al.*, 2019). Tests such as PCA and network analysis were primarily conducted to understand the covariation and network connections between generally unknown traits such as SPA and PDMC with well-established traits such as SLA and LDMC.

To assess the covariation of plant traits in response to elevated CO_2_, increased temperature and drought, separate PCAs were conducted for each treatment. Because the number of individuals per treatment was unbalanced, we performed multiple independent tests using repeated random sampling to standardize the dataset. In each iteration, we randomly selected five individuals per treatment for *L. corniculatus* and six individuals per treatment for *C. capillaris*, ensuring equal sample sizes across treatments. This repeated subsampling approach allowed us to assess the robustness of trait covariation patterns across treatments. The significance of the resulting PCAs and the contribution of individual traits to the significant axes were evaluated using the PCAtest package version 0.0.2 (Camargo, 2022). The proportion of variance explained by the PCA axes for each species was visualized as rank–abundance curves using the ggplot2 package (Wickham, 2016).

## RESULTS

The results are presented in three subsections. The first section details the responses of traits of both plant species to the individual and interactive effects of climate change factors. For each species, we first report findings from the multivariate analysis, followed by results from the univariate analyses. The second section examines the covariation among plant traits, while the final section presents how trait covariation is influenced by climate change factors.

### Response of plant functional traits to climate change factors

#### Lotus corniculatus

**Fig. 1.**
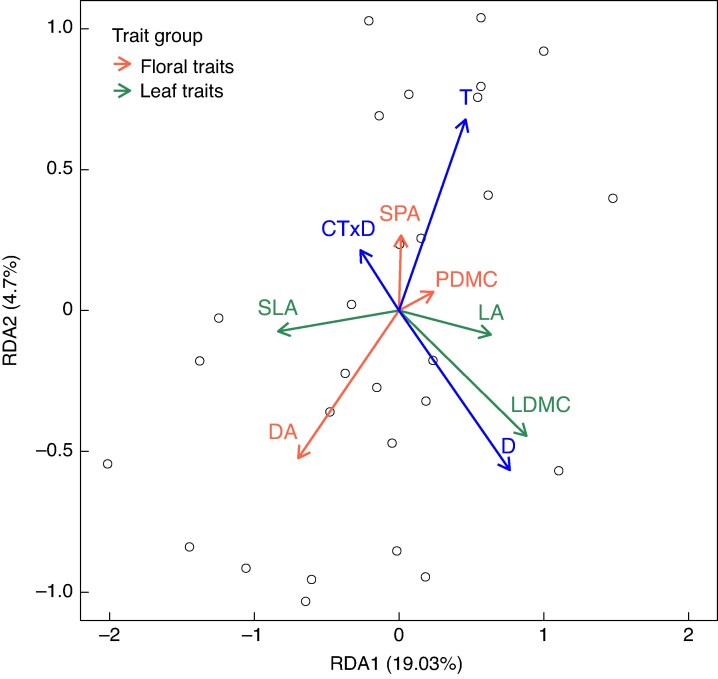
RDA plot of *L. corniculatus*. Significant climate change factors are shown in blue while plant traits are coloured based on their group (as shown in the key). T, warming (+3 °C); D, drought; CT × D, elevated CO_2_, warming and drought.

The three climate change factors and their interactions collectively explained 29 % of the trait variation in the multivariate RDA (*P* = 0.002 for the overall model). When tested individually, drought (D) and warming (T) had significant effects and CT × D had marginally significant effects (Fig. 1), while elevated CO_2_ and C × T did not have any significant effect. Among the significant predictors, drought accounted for the highest variance (10.2 %), followed by warming (6.3 %) and then CT × D (4.8 %) (Supplementary Data Table S2).

Drought and temperature were the primary drivers of variation along the first RDA axis, which accounted for 19.03 % of the variation in trait responses. Display area and leaf traits (LA, SLA and LDMC) varied along this gradient, with increasing drought being associated with an increase in LA and LDMC alongside a decrease in SLA. Temperature was diametrically opposite to DA, indicating that increasing temperature levels would lead to a reduction in DA. The second RDA axis accounted for 4.7 % of trait variation and was related to CT × D. Display area was well represented on this axis, indicating that the most extreme condition, CTD, would lead to a decrease in DA (Details on Trait Loadings have been provided in Supplementary Data Table S6).

**Fig. 2.**
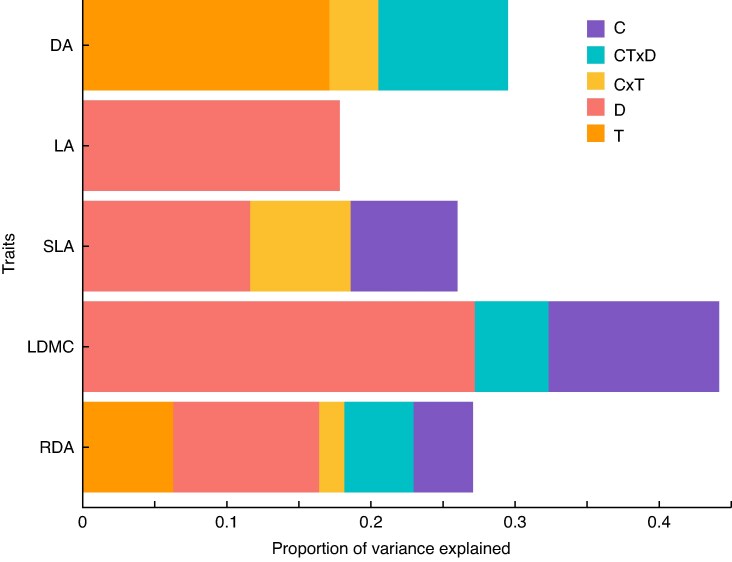
Variance partitioning plot for *L. corniculatus* showing the magnitude of variation explained by the different climate change factors. C, elevated CO_2_ (+300 ppm); T, warming (+3 °C); D, drought; C × T, elevated CO_2_ combined with warming; CT × D, elevated CO_2_, warming and drought. RDA results are of multivariate analysis. Only the significant (*P* < 0.05) variables are shown. SPA and PDMC do not have any significant predictors and are thus not shown.

**Fig. 3.**
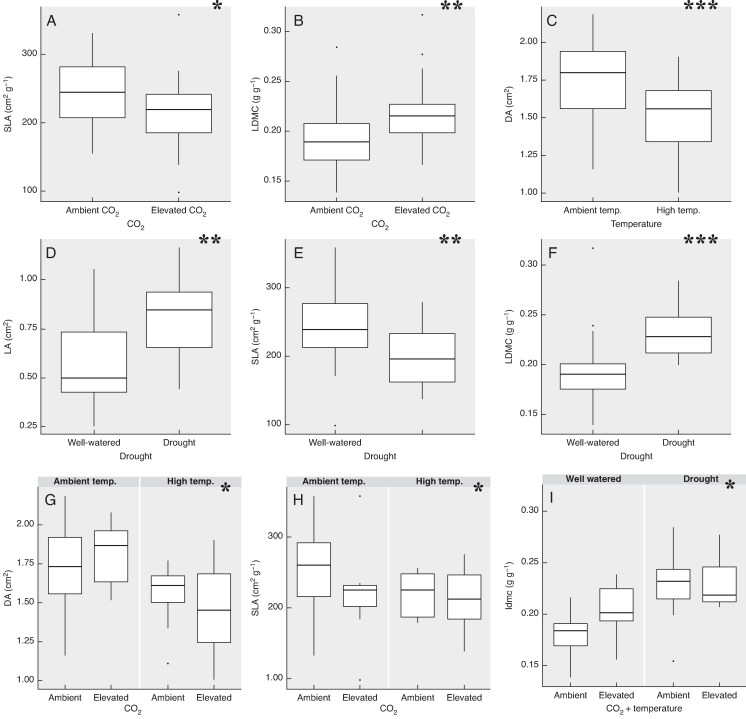
Box plots illustrating the individual and combined effects of climate change factors on plant traits of *L. corniculatus*. Only significant effects are shown; the significance level of the effect of the plotted factor or their interaction is indicated in the top right corner (*P = 0.05; **P = 0.01; ***P ≤ 0.001). The functional trait studied is denoted on the *Y*-axis while treatment is denoted on the *X*-axis.

The univariate analyses of *L. corniculatus* traits revealed that drought had the most consistent and significant effects (Fig. 2), increasing LA and LDMC while decreasing SLA (Fig. 3D–F). CO_2_ induced variation in two out of the three leaf traits, reducing SLA and increasing LDMC (Fig. 3A, B). The temperature effect was more trait-specific as it had a significant effect only on DA (Fig. 3C), causing a substantial decrease in DA under higher temperature. In terms of interactions, C × T overall caused a decrease in DA, as warming negated and reversed the positive effect of CO_2_ (Fig. 3G). In the case of SLA, the decrease caused by CO_2_ became stagnant with increasing temperature (Fig. 3H). In the most extreme treatment (CT × D), drought had a buffering effect on the increase in LDMC by CO_2_ and temperature (Fig. 3I). *P*-values of Significant Effects Detailed in Table 1.

**Table 1.** Effect of climate change factors (CO_2_, temperature and drought) on plant traits of *L. corniculatus* and *C. capillaris.*

		Treatment										
			C	T	D	CT	CTD	C	T	D	CT	CTD
		*L. corniculatus*						*C. capillaris*				
Flower	Display area	*F*	2.29	**21**.**49**	0.12	**4**.**26**	**11**.**25**	1.91	0.01	**5**.**05**	0.02	0.07
		*P*	0.134	**< 0.001**	0.729	**0**.**042**	**0**.**001**	0.174	0.942	**0**.**030**	0.900	0.788
	Specific petal area	*F*	0.06	0.67	3.24	0.52	0.21	0.27	0.35	**8**.**06**	*3*.*05*	1.22
		*P*	0.808	0.414	*0*.*075*	0.474	0.647	0.607	0.560	**0**.**007**	*0*.*088*	0.275
	Petal dry matter content	*F*	0.01	1.30	1.08	0.05	0.29	0.17	0.19	**9**.**76**	*3*.*19*	2.64
		*P*	0.931	0.257	0.301	0.815	0.594	0.679	0.667	**0**.**003**	*0*.*081*	0.111
Leaf	Leaf area	*F*	1.08	3.31	**10**.**86**	0.29	0.34	0.00	0.00	0.22	0.25	0.60
		*P*	0.305	*0*.*076*	**0**.**002**	0.596	0.561	0.948	0.980	0.644	0.619	0.445
	Specific leaf area	*F*	**4**.**96**	2.23	**7**.**76**	**4**.**63**	2.14	0.00	0.00	0.22	0.25	0.60
		*P*	**0**.**031**	0.143	**0**.**008**	**0**.**037**	0.151	0.948	0.980	0.644	0.619	0.445
	Leaf dry matter content	*F*	**9**.**76**	0.05	**22**.**44**	1.00	4.21	0.10	0.06	**5**.**89**	1.17	2.98
		*P*	**0**.**003**	0.816	**< 0.001**	0.322	**0**.**046**	0.751	0.813	**0**.**020**	0.287	0.092
Seed	Seed number	*F*						0.92	**5**.**31**	**12**.**66**	*3*.*52*	0.30
		*P*						0.344	**0**.**027**	**0**.**001**	*0*.*068*	0.588
	Seed mass	*F*						0.51	1.51	0.42	2.08	0.01
		*P*						0.478	0.227	0.520	0.157	0.939

Significant effects (*P*-values < 0.05) are marked in bold while marginally significant effects (*P*-values between 0.05 and 0.1) are marked in italics.

C, elevated CO_2_; T, warming; D, draught; CT elevated CO_2_ and warming; CTD, elevated CO_2_, warming and drought.

#### Crepis capillaris

**Fig. 4.**
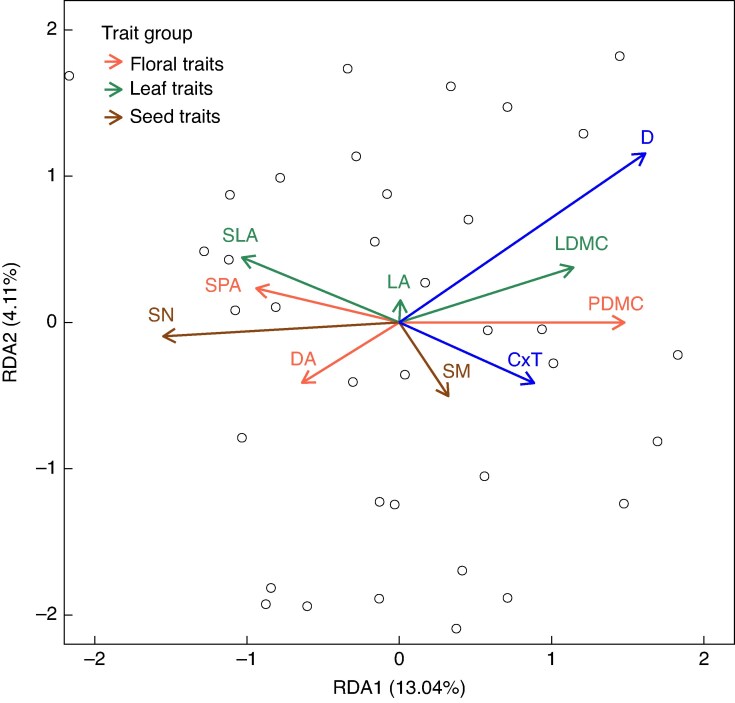
RDA plot of *C. capillaris*. Significant climate change factors are shown in blue while plant traits are coloured based on their group (as shown in key). D, drought; T, warming; CT, elevated CO_2_ + warming; C × T, elevated CO_2_ combined with warming.

The RDA analysis of *C. capillaris* showed that the climate change factors accounted for 20 % of the total trait variation (*P* = 0.003 for the overall model), and drought (7.7 %) and C × T (4.5 %) had significant effects on the plant traits (Fig. 4).

These two climate change factors were highly correlated in the first RDA axis (13 %), with drought being the primary and C × T the secondary contributor to variation along this axis. The constrained variation in floral traits (DA, SPA and PDMC), leaf traits (SLA and LDMC) and SN was chiefly represented along this axis. The patterns indicated that drought had a positive relationship with the dry matter content traits (LDMC and PDMC) while having a negative relationship with all other traits. Among these traits, SN showed the strongest inverse relationship with drought. The second RDA axis accounted for 4 % of the total variation, with temperature as the dominant factor. Leaf area was best represented along this axis, possibly indicating that increasing temperature would cause a decrease in LA, although this relationship was not significant (Details on Trait Loadings have been provided in Supplementary Data Table S7).

**Fig. 5.**
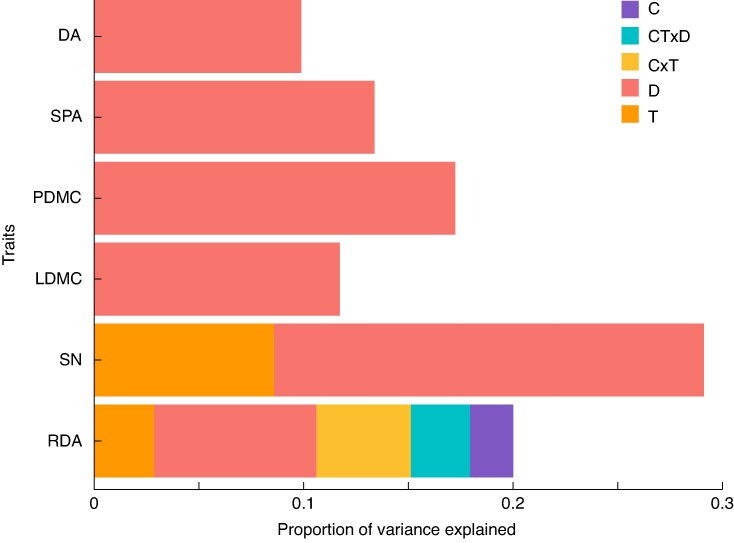
Variance partitioning plot for *C. capillaris* explaining magnitude of variation in relation to the three climate change factors and their interactions. C, elevated CO_2_ (+300 ppm); T, warming (+3 °C); D, drought; C × T, elevated CO_2_ combined with warming; CT × D, elevated CO_2_, warming and drought. RDA results are of multivariate analysis. Only the significant (*P* < 0.05) variables are shown. Specific leaf area does not have significant predictors.

**Fig. 6.**
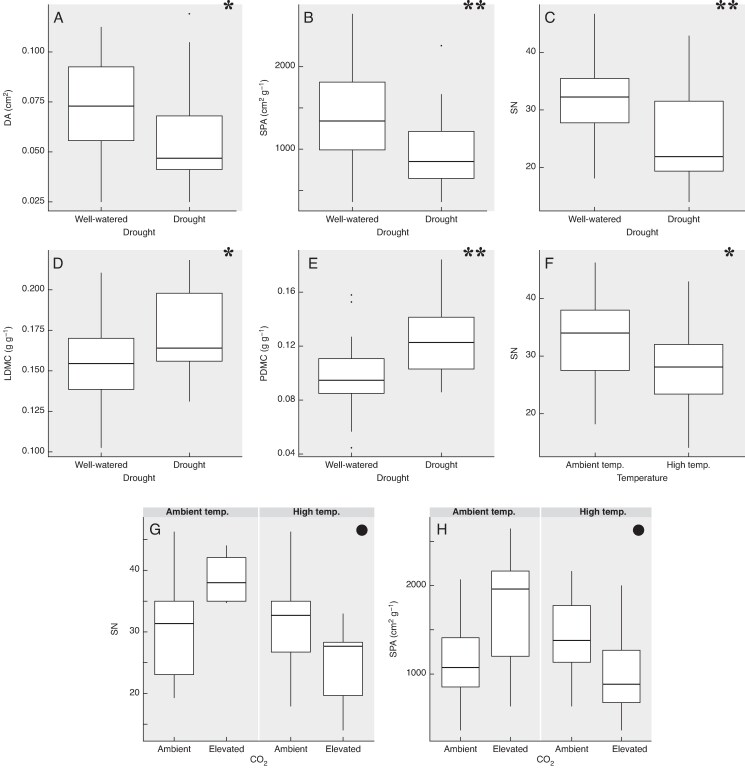
Box plots illustrating the effects of climate change factors on plant traits of *C. capillaris*. Only the significant effects have been shown; the significance level of the effect of the plotted factor or their interaction is indicated in the top right corner (●*P* = <0.1; *P = 0.05; **P = 0.01). The functional trait studied is denoted in the *Y*-axis while treatment is denoted in the *X*-axis.

The findings in the univariate analysis reflected the general patterns observed in the RDA. Similar to *L. corniculatus*, trait variation in *C. capillaris* was primarily dictated by drought, which was the only climatic variable having a significant effect on the majority of plant traits. Its magnitude of variation was highest in SN, followed by PDMC and then the other traits (SPA, LDMC and DA) (Fig. 5). An increase in drought levels caused a significant decrease in DA, SPA and SN while leading to an increase in PDMC and LDMC (Fig. 6A–E)(P-values of Significant Effects Detailed in Table 1).

Similar to *L. corniculatus*, the effect of temperature in *C. capillaris* was also trait-specific and caused a significant effect only for SN, which was negatively related to warming (Fig. 6F). In terms of interactions, although under ambient temperature conditions an increase in CO_2_ intensity caused an increase in SPA, warming was shown to negate the positive effect of CO_2_ with CT, showing a slight reduction in SPA despite the positive effect of CO_2_ (Fig. 6G). This pattern is mirrored in the effect of CO_2_ and temperature on SN (Fig. 6H).

### Covariation between plant traits

**Fig. 7.**
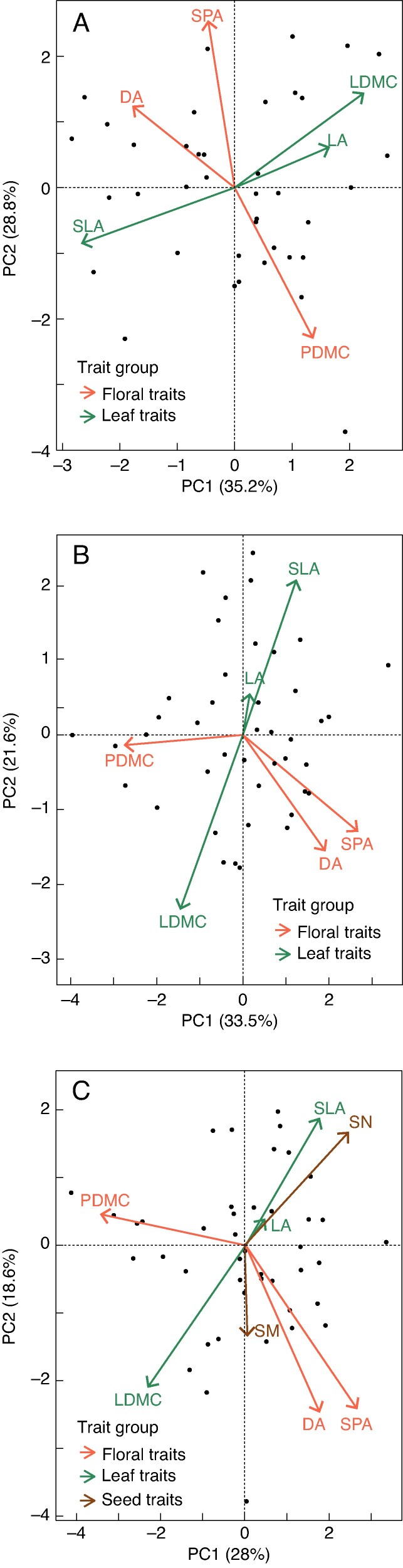
PCA plot of (A) *L. corniculatus*, (B) *C. capillaris* (without seed traits) and (C) *C. capillaris* (with seed traits).

The initial PCA of both species revealed two independent axes of variation among leaf and floral traits. In *L. corniculatus*, the first two axes accounted for 64 % of the variation (Fig. 7A). The first axis accounted for 35.2 % of the variation and was primarily associated with leaf traits (SLA and LDMC). The second axis accounted for 28.8 % of the variation and was linked to floral traits (SPA and PDMC). Specific area traits (SLA and SPA) were negatively correlated with their dry matter counterparts (LDMC and PDMC). Regarding floral traits, DA occurred close to SPA, and together they were orthogonal to the leaf traits, where LA occurred close to LDMC (Trait Loadings have been detailed in Supplementary Data Table S4).

In *C. capillaris*, the first two principal components accounted for 55.2 % of the total trait variation (when analysed without seed traits) (Fig. 7B). The first axis was dominated by floral traits (DA, SPA and PDMC) (33.6 %), while the second axis was dominated by leaf traits (LA, SLA and LDMC). Similar to DA in *L. corniculatus*, DA in *C. capillaris* occurred close to SPA and was orthogonal to leaf traits (SLA and LDMC). Specific leaf area and LDMC were also negatively correlated (Trait Loadings have been detailed in Supplementary Data Table S5).

When seed traits (SN and SM) were added to the PCA (Fig. 7C), the overall patterns of dominance remained the same. Seed number was negatively correlated with the dry matter content traits (PDMC and LDMC). The loadings for SM consistently had low absolute values across all principal components, with a maximum of −0.3 on PC3. This implied that SM played a negligible role in defining the principal components.

**Fig. 8.**
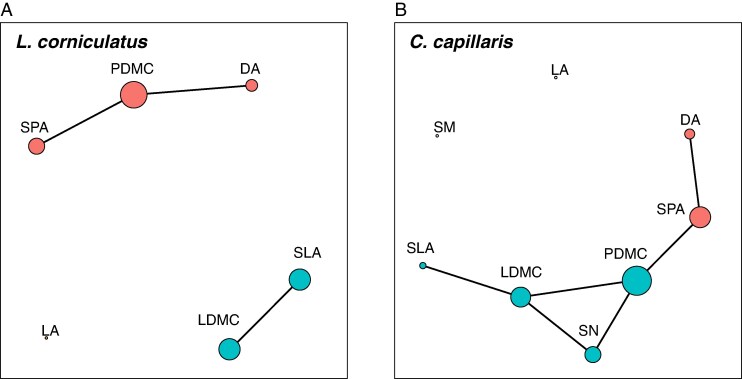
Trait correlation network for (A) *L. corniculatus* and (B) *C. capillaris*. Node colours indicate separate clusters or ‘modules’ while node size shows betweenness. Correlation strength is shown by line thickness and distance between traits.

Network analysis of the two species confirmed the findings from the PCAs while offering some additional insights by revealing the strength and structure of trait interrelationships. In *L. corniculatus*, the most connected and most central trait was PDMC (Fig. 8A), which connected the other floral traits, SPA and DA while having a stronger connection with the former (Supplementary Data Table S3). Both floral and leaf traits independently clustered with the leaf traits, having stronger interactions among each other than the floral traits (Fig. 8). These findings contrast with those for *C. capillaris* (Fig. 8B), where dry matter content traits, specifically LDMC and PDMC, formed a cluster alongside SLA and SN. Specific petal area and PDMC dominated the network both in degree and betweenness. Traits such as LA and SM did not play any role in the network.

### Coordinated changes in traits in response to climate change factors

**Fig. 9.**
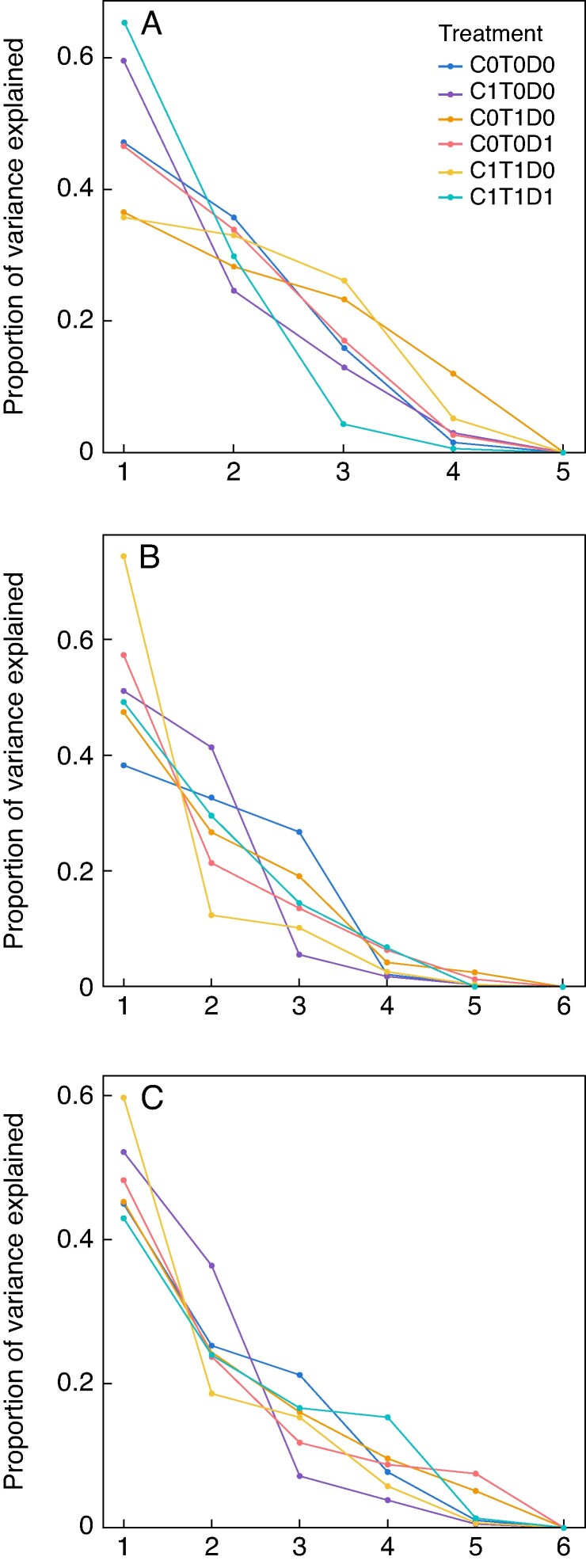
Variation explained in the traits by each of the PCA axes under different treatments in (A) *L. corniculatus*, (B) *C. capillaris* (without seed traits) and (C) *C. capillaris* (with seed traits). C0T0D0, ambient control; C1T0D0, elevated CO_2_ (+300 ppm); C0T1D0, warming (+3 °C), C0T0D1, drought; C1T1D0, elevated CO_2_ combined with warming; C1T1D1, elevated CO_2_, warming and drought.

When analysing the covariation of plant traits under each climatic regime separately, we found that different treatments led to coordinated adjustments in traits. However, the coordinated responses were not similar across species. In *L. corniculatus*, the most extreme treatment (C1T1D1) caused a major coordinated adjustment in traits (Fig. 9A), with PC1 being the only significant axis, explaining 65.3 % of the total variation. Variation in this axis was dictated by SPA and LDMC. Traits in the other treatments did not show any significant covariations.

These patterns were partially mirrored in *C. capillaris* (Fig. 9B). Under the combined elevated CO_2_ and warming treatment, PC1 accounted for 74.5 % of the total trait variance and was statistically significant. Specific petal area, PDMC, LA and SLA all had strong loadings on PC1, with SPA, LA and SLA also showing significant correlations with the axis. In contrast, under the elevated CO_2_ treatment alone, only PC2 was significant, explaining 41.4 % of the variance. In this case, LA and LDMC loaded significantly on PC2, with LDMC also exhibiting a significant correlation with the axis. No significant trait covariation was observed in the remaining treatment combinations. These PCA analyses should be interpreted with caution as the small sample size means that the number of variables analysed was equal to or greater than the number of observations. This increases the risk of overfitting and reduces the robustness of the PCA outcomes. To mitigate this limitation, we interpreted only the treatments that consistently showed statistical significance across multiple randomizations. This strategy helped ensure that the observed patterns of trait covariation were not artefacts of sampling variability, but reflected biologically meaningful responses, albeit with caution with regard to their generalizability.

## DISCUSSION

The results of this study show that climate change simulated for multiple years significantly impacts not only vegetative traits but also reproductive traits such as floral and seed traits, although responses vary between species. Our study supported hypothesis 1, indicating that plant traits respond to drought more consistently than to elevated CO_2_ and warming. Contrary to hypothesis 2, the negative effects of temperature were not mitigated by elevated CO_2_ conditions. Finally, in line with hypothesis 3, climate change factors and their interactions exerted a strong filtering on covariation of plant traits, albeit this phenomenon was species-specific and our data have limited strength to prove this unequivocally.

### Response of plant functional traits to individual climate change factors

Climate change factors significantly influenced the functional traits of both *L. corniculatus* and *C. capillaris*, though the responses varied across traits and species. In *L. corniculatus*, drought was the dominant factor, but other climate change factors also affected two of the three leaf traits (SLA and LDMC) and one floral trait (DA). In *C. capillaris*, drought played a major role, significantly impacting all floral traits (DA, SPA and PDMC), as well as one leaf trait (LDMC) and one seed trait (SN) (Significance of Individual Plant Traits hav been provided in Supplementary Data Table S9). Temperature was the only other significant factor, affecting only SN. Species-specific responses have been reported in many previous studies (Ainsworth and Long, 2005; Descamps *et al.*, 2020; Kuppler and Kotowska, 2021; Bernauer *et al.*, 2024).

The finding that drought is the dominant factor affecting economic traits of both species aligns with our hypothesis 1. Drought stress has been consistently shown to reduce traits such as LA, SLA, flower size and SN (Lidon, 2012; Wellstein *et al.*, 2017; Matesanz *et al.*, 2020; Descamps *et al.*, 2021*a*; Höfer *et al.*, 2023), while increasing LDMC (Jung *et al.*, 2014). This reflects a shift towards more carbon-demanding structures with higher construction costs per unit area. A notable exception in our study is the response of LA. In *L. corniculatus*, LA increased under drought, contrary to what was found by most prior studies (e.g. Westoby *et al.*, 2002; McDonald *et al.*, 2003; Yates *et al.*, 2010; Bjorkman *et al.*, 2018; Zhou *et al.*, 2020). Our finding is supported by a study of *Limonium* species, where larger leaves, despite being considered more carbon-demanding in terms of construction cost, were associated with higher growth capacity and higher water use efficiency (Conesa *et al.*, 2019). Our study demonstrated a more conservative strategy involving higher carbon investment, in which plants increased dry matter content traits (such as LDMC and PDMC) while reducing tissue construction cost-related traits (such as SLA and SPA). This shift suggests a higher investment in structural tissues, aligning with the plant’s adaptive response to environmental stress (Poorter *et al.*, 2009). These tissues, although more costly to produce, are more durable and enhance plant resistance under environmental stress (Gong *et al.*, 2020; Wang *et al.*, 2022; Akram *et al.*, 2023). Smaller plant organs generally require less water to maintain turgor pressure, which can improve plant water status during drought stress by reducing overall transpiration and hydraulic demand (Galen *et al.*, 1999; Chaves *et al.*, 2002). Although this strategy is cost-effective for temporary survival, the reduction in flower size, a crucial visual cue for pollinators, could potentially decrease pollinator visitation rates and thereby reduce population growth rates (Descamps *et al.*, 2021*b*). However, a decrease in SPA may also suggest increased floral longevity due to reduced flower maintenance costs (Spigler and Woodard, 2019). This could mitigate the potential negative effects of smaller flower size. While no general patterns of decreasing visitation rates by pollinators under water deficit conditions have been identified (reviewed in Kuppler and Kotowska, 2021), the potential impact of floral trait variation on pollinator behaviour requires further attention.

Warming influenced only two plant traits: it reduced floral DA in *L. corniculatus* and SN in *C. capillaris*, without affecting any resource economic traits. Although there are many previous studies that predicted a significant linear relationship between leaf traits (LA, SLA and LDMC) and temperature (e.g. Atkin *et al.*, 2006; Poorter *et al.*, 2010; Fontana *et al.*, 2017; Dostálek *et al.*, 2020), our findings on leaf traits were more in line with a study conducted in the Bavarian Alps consisting of 223 species (Rosbakh *et al.*, 2015), where a significant relationship between SLA and temperature was found only at the community level, with no response at either the species or population level. This could be because of the predominant effect of drought on vegetative traits as water availability is a greater limiting factor than temperature in temperate climates (Fahad *et al.*, 2017; Zhou *et al.*, 2017). In regard to floral traits, although warming did not alter the per area structural investment in either species, it led to reduced DA in one of them. This response may be attributed to the greater sensitivity of reproductive development to abiotic stresses compared with vegetative growth (Zinn *et al.*, 2010; Snider and Oosterhuis, 2011). Under warming conditions, this heightened sensitivity can lead to reductions in floral organ size, potentially through altered expression of genes in floral morphogenesis (Zinn *et al.*, 2010; Smith and Zhao, 2016). In light of this, species capable of maintaining and increasing floral display size under elevated temperatures have a greater probability of surviving in future climate scenarios over species which cannot. However, this strategy may involve trade-offs between display size and floral longevity, as larger flowers typically require greater construction costs and may have shorter lifespans (Reich, 2014). Plants that can flexibly adjust their display size and flower longevity can enhance pollinator attraction when pollinators are rare by increasing their display size and limit self-pollination when pollinators are common by reducing their display size (Harder and Johnson, 2005). These dynamics highlight the importance of floral trait plasticity – not just in size but also in timing and lifespan – as a potential adaptive response to climate change. While our study did not include measurements of flower longevity, incorporating this trait in future research would provide valuable insights into how plants balance reproductive investment under environmental stress.

In our experiment, elevated CO_2_ levels resulted in a decrease in SLA and increase in LDMC in only one species (*L. corniculatus*). Similar to the drought response, this pattern suggests a shift towards a more carbon-demanding and conservative strategy. While several studies confirm our findings of decrease in SLA and increase in LDMC under elevated CO_2_ (e.g. Centritto *et al.*, 1999; Stiling and Cornelissen, 2007; Robinson *et al.*, 2012; Poorter *et al.*, 2022), others partially contradict them, showing increased SLA under elevated CO_2_ (Duan *et al.*, 2018; Temme *et al.*, 2019). This variation may be due to differences in resource-use strategies among plants with different growth forms. Perennials like *L. corniculatus* often have thicker leaves with lower SLA and higher LDMC, leading to slower growth rates and lower resource acquisition compared with faster-growing annuals (Poorter *et al.*, 2009; Reich, 2014). When exposed to favourable conditions (such as elevated CO_2_), perennials may not exhibit the same growth responses as annuals because their strategies prioritize survival and maintenance over rapid growth. In our study, elevated CO_2_ level had no effect on floral traits. This is in line with Bernauer *et al.* (2024), while other studies have reported both increase and decrease of flower size under elevated CO_2_ (Niu *et al.*, 2000; Hoover *et al.*, 2012; Maia *et al.*, 2023). Overall, our findings highlight how environmental stressors such as drought and elevated CO_2_ drive shifts in plant economic strategies. Traits like SLA and SPA serve as indicators of tissue construction cost, with lower values reflecting more carbon-demanding and more structurally robust tissues. Conversely, higher dry matter content traits (LDMC and PDMC) signal a conservative strategy prioritizing durability over rapid resource acquisition. These shifts underscore the importance of integrating plant economics frameworks when interpreting trait responses to climate change.

### Interactive effects of climate change factors on plant traits

Regarding the combined effects of climate change factors on plant traits, we hypothesized (hypothesis 2) that the negative effects of either warming or drought would be partially mitigated by elevated CO_2_. This hypothesis was not supported by our observations. The combination of elevated CO_2_ and warming reduced DA of *L. corniculatus*, and SPA and SN in *C. capillaris*. Similar findings have been reported in other studies. For instance, Li *et al.* (2019), in their study on *Morus alba*, found that elevated CO_2_₂ and temperature together reduced the number and biomass of male inflorescences, while positively affecting female inflorescences. Notably, the fresh weight of male inflorescences under combined elevated CO_2_ and warming was lower than under warming alone, mirroring the pattern observed in our study (Li *et al.*, 2019). Other research has also shown that elevated CO_2_ does not offset the negative effects of warming on seed yield, likely due to reduced photosynthetic efficiency under heat stress, which can lead to ovule abortion and impaired reproductive development (Ruiz-Vera *et al.*, 2015; Notarnicola *et al.*, 2023).

The combination of elevated CO_2_, warming and drought (CT × D) significantly affected only one economic trait in only one species, i.e. LDMC in *L. corniculatus*. While plants exposed to elevated CO_2_ and temperature exhibited higher LDMC than those in ambient conditions, drought-stressed plants showed a significantly greater LDMC than both ambient and elevated CO_2_ + temperature. Increased LDMC might be attributed to improved water-use efficiency, driven by reduced SLA (Wellstein *et al.*, 2017) and increased investment in non-structural carbohydrates (Blumenthal *et al.*, 2020; Rodríguez-Alarcón *et al.*, 2022). Changes in palisade parenchyma cell composition (specifically increases in cell number and size under drought) may also contribute to reduced SLA and increased LDMC (Peña-Rojas *et al.*, 2024), reinforcing the link between anatomical adaptation and economic trait shifts.

### Trait covariation patterns and effect of climate change factors on covariations

Our findings on trait covariation patterns reveal that floral traits of *L. corniculatus* are largely independent of leaf traits, and this is in line with E-Vojtkó *et al.* (2022). In *C. capillaris*, however, there seems to be an interesting link between PDMC and LDMC, with both traits also having SN as a mediator. Although previous studies do not specifically use these two traits, overall plant dry matter has been found to be positively correlated with SN (Gerik *et al.*, 2004). This would be due to the higher dry matter accumulation enabling plants to store and mobilize more resources for seed development, leading to increased seed yield (Liu *et al.*, 2020). In regard to the effects of climate change factors on trait covariation, we hypothesized (hypothesis 3) that environmental extremes exert a strong coordinated response in plant functional traits. This phenomenon was found to be true for only one of our two species. This differential coordinated response of plant functional traits may reveal interesting insights into the adaptability and distribution of plant species across different habitats.

Functional traits of *L. corniculatus* showed the most coordinated response in the most extreme conditions (C1T1D1). This is in line with previous studies (Lozano *et al.*, 2020; Mueller *et al*, 2024). Drier, warmer habitats have been found to exert stronger selection pressures on plant traits to enhance water-use efficiency, heat tolerance and resource conservation, which eventually leads to a stronger selection for trait coordination (Blumenthal *et al.*, 2020; Mueller *et al.*, 2024). *Crepis capillaris* showed coordinated trait responses primarily under elevated CO_2_ alone (C1T0D0) and in combination with elevated temperature (C1T1D0). The absence of a coordinated trait response to drought in *C. capillaris*, unlike in *L. corniculatus*, may contribute to the differences in their ecological distribution and adaptive capacity. *Lotus corniculatus* is widely distributed across diverse climatic zones, thriving in temperate, tropical and subtropical climates while also occurring in certain regions of the polar and sub-polar zones. Although *C. capillaris* is also broadly distributed, it is primarily restricted to the temperate regions of Europe and North America while having some coverage in the subtropical zones of Africa and Australia (Plants of the World Online, 2024). These findings suggest that trait coordination under stress may support ecological adaptability and range expansion. However, it is unlikely to be the sole factor determining species distribution, which is also shaped by other ecological and physiological constraints.

Overall, our results demonstrate that climate change factors exert strong filtering effects on plant trait coordination, although the nature of these responses varies across species. Previous work has highlighted increased trait coordination along environmental gradients such as rainfall (Li *et al.*, 2018) and salinity (Bricca *et al.*, 2023). The novelty of our study lies in examining these dynamics within a controlled environment across multiple climate change drivers, offering mechanistic insights into species-specific responses to future climate scenarios.

### Conclusions

Our study highlights the significant impact of individual and interactive effects of climate change factors, including CO_2_, temperature and drought, on the floral economic traits of *L. corniculatus* and *C. capillaris*. Drought emerged as the dominant factor influencing most plant traits, with species-specific variations in response intensity. The interactive effects of these climate change factors also led to varied responses in floral economic traits. While both species exhibited coordinated trait responses under combined treatments, *C. capillaris* did not show a coordinated response to drought, potentially contributing to its more restricted distribution. To gain a comprehensive understanding of resource allocation strategies (trade-offs among traits) in plants under environmental stresses, larger studies encompassing a broader range of functional traits and larger species set are essential. Investigating the floral economic spectrum and its integration into the whole plant economic spectrum can provide valuable insights into the effects of divergent selective forces on different plant tissues and the potential consequences of shifts in these selective forces as a result of climate change.

## Supplementary Material

mcaf214_Supplementary_Data

## Data Availability

The datasets and R scripts supporting the findings of this study are available in Zenodo at https://doi.org/10.5281/zenodo.17360691.

## SUPPLEMENTARY DATA

Supplementary data are available at *Annals of Botany* online and consist of the following. Figure S1: PCA plots showing trait coordination patterns of *L. corniculatus* under different treatments. Figure S2: PCA plots showing trait coordination patterns of *C. capillaris* under different treatments (without seed traits). Figure S3: PCA plots showing trait coordination patterns of *C. capillaris* under different treatments (with seed traits). Table S1: numbers of individuals and plots from which samples of each treatment of *L. corniculatus* and *C. capillaris* were collected. Table S2: effect of climate on trait composition tested using RDA on *L. corniculatus* and *C. capillaris*. Table S3: trait degree (number of edges) and betweenness centrality in the network, weighted by correlation strength. Table S4: trait loadings and percentages of trait variation explained by successive principal components (PCs) in *L. corniculatus*. Table S5: trait loadings and percentages of trait variation explained by successive principal components (PCs) in *C. capillaris*. Table S6: loadings of traits and significance of successive constrained axes in RDA in *L. corniculatus*. Table S7: loadings of traits and significances of successive constrained axes in RDA in *C. capillaris*. Table S8: dates of induced drought at ClimGrass experimental plots. Table S9: ecological significance of the studied plant traits.

## AI ASSISTANCE

AI assistants, including ChatGPT and Copilot, were utilized to aid in literature gathering, assist with statistical analysis, and refine the tone of the Introduction and Discussion sections. All outputs were meticulously cross-checked by the authors to ensure accuracy and prevent the inclusion of false information in the manuscript.
