# Correction to: Elevated CO_2_, warming and drought differentially impact reproductive and vegetative economic traits in two grassland species

**DOI:** 10.1093/aob/mcag119

**Published:** 2026-05-16

This is a correction to: Murugash Manavalan, Dinesh Thakur, Andreas Schaumberger, Michael Bahn, Zuzana Münzbergová, Elevated CO_2_, warming and drought differentially impact reproductive and vegetative economic traits in two grassland species, *Annals of Botany*, Volume 137, Issue 3, March 2026, Pages 673–688, https://doi.org/10.1093/aob/mcaf214

In the originally published version of this manuscript, an institutional affiliation was missing for corresponding author Murugash Manavalan.

This author's affiliation to “Institute of Botany, Academy of Sciences of the Czech Republic, Průhonice 252 43, Czech Republic” has now been added.

